# Is the level of institutionalisation found in psychiatric housing services associated with the severity of illness and the functional impairment of the patients? A patient record analysis

**DOI:** 10.1186/s12888-015-0595-6

**Published:** 2015-09-14

**Authors:** Juan Valdes-Stauber, Reinhold Kilian

**Affiliations:** Department of Psychiatry and Psychotherapy I, University of Ulm at Zentrum für Psychiatrie Südwürttemberg, Ravensburg, Germany; Department of Psychiatry and Psychotherapy II, Department of Psychiatry and Psychotherapy II of the University of Ulm at Bezirkskrankenhaus Günzburg, Günzburg, Germany

## Abstract

**Background:**

In this cross-sectional study, we investigated whether clinical, social, financial, and care variables were associated with different accommodation settings for individuals suffering from severe and persistent mental disorders.

**Method:**

Electronic record data of 250 patients who fulfilled the criteria for persistent and severe mental illness were used. Multiple linear regression models were applied to analyse associations between the types and the costs of housing services and the patients’ severity of illness, their functional impairment, and their socio-demographic characteristics.

**Results:**

We identified 50 patients living at home without need for additional housing support who were receiving outpatient treatment, 41 patients living in the community with outpatient housing support, 23 patients living with foster families for adults, 45 patients living in group homes with 12-h staff cover, 10 patients living in group homes with 24-h staff, and 81 patients living in psychiatric nursing homes. While this housing differed largely in the level of institutionalisation and also in the costs of accommodation, these differences were not related to a patient’s severity of disease or in their functional impairment. In particular, patients living in nursing homes had a slightly higher level of functioning compared to those living in the community without welfare housing services. Only where patients were subject to guardianship was there a significant association with an increased level of institutionalisation.

**Conclusions:**

Our study suggests that the level of institutionalisation and the associated costs of welfare housing services do not accurately reflect the severity of illness or the level of functional impairment of the patients there are designed to support. The limitations of the study design and the data do not allow for conclusions about causal relationships or generalisation of the findings to other regions. Therefore, further prospective studies are needed to assess the adequacy of the setting assignment of patients with persistent severe mental illness into different types of housing settings with appropriate (also welfare) services.

## Background

In contrast to traditional psychiatric institutions, contemporary systems of housing support for people with severe mental disorders were envisaged to offer a broad variety of services, tailored as closely as possible to the individual needs of a particular patient. Such services range from assisting people to live in their own home to the provision of full-time care in psychiatric nursing facilities [1]. Importantly, support services should be able to respond adequately to changes in patients’ needs. This implies that, depending on their current level of impairment, the users of residential services can easily switch between different levels of care.

Currently, there is limited information on the extent to which these expectations have been realized in Europe [2]. Priebe et al. [3, 4] show that between 1990 and 2006, several European countries significantly increased the number of residential care facilities. The authors discuss whether this rise indicates a general trend in the direction of reinstitutionalisation of psychiatric care and highlight the fact that most European countries still have no clear criteria for assessing the quality of residential care facilities.

In Germany, the number of residential facilities for people with mental illness increased from 8.9 per 100,000 persons in 1990 to 63.3 per 100,000 persons in 2006 [3, 4]. Recent data indicate that in 2010 48,682 patients with mental illness lived in psychiatric nursing homes whilst 81,094 were in outpatient housing service settings [5, 6]. The overall ratio between residential and outpatient housing services in Germany is 0.6 but that ratio varies significantly among the federal states from 0.09 to 2.42. (National Working Committee of Supreme Public Health Authorities of German States, 2014). The total costs of residential care for mentally-ill people [5, 6] in the year 2010 amounted to 7.5 billion Euros [7].

To date the relationships between the type of housing service and severity of disease or functional impairment have only been investigated in a small number of studies. In Italy, de Girolamo et al. [8] examined a sample of 2962 residents from 265 facilities and found that the level of care measured by staff hours was positively related to the level of impairment measured by the Global Assessment of Functioning Scale (GAF) and the Health of Nations Outcome Scale (HoNOS). However, they also found that the chance of the residents being discharged from their current residential facility and switching to a facility with a lower level of institutionalisation was rather small and difficult to predict on the basis of the individual level of impairment [8, 9]. In the UK, Lelliott et al. [10] examined 1951 residents of 368 facilities and found a positive association between the severity of resident disability and the level of staffing in the residential settings. Also in the UK, a more recent study of 414 patients from 183 residential services by Priebe et al. found that the level of institutionalisation (home care vs. supported housing vs. floating support) was positively related to the number of met service needs assessed by the Camberwell Assessment of Needs (CAN) but that the characteristics of clients greatly overlapped between the different types of services [11].

In the Netherlands, de Heer-Wunderink et al. [12] compared the characteristics of 1656 residents in Dutch residential facilities with those of the residents from Italy examined by de Girolamo et al. They found that Dutch users of residential facilities were younger and had a higher level of functioning than those from Italy. The authors concluded from these results that the de-institutionalisation process in the Netherlands was not as advanced as in Italy [12]. In another study, the same authors compared the characteristics of 534 Dutch mentally-ill residents with those of the UK sample examined by Priebe et al. [11]. They found that Dutch residents in assisted housing and users of supported independent living services had more met service needs that those of the UK sample. Moreover, the comparison of the met service needs and the clinical characteristics of the Dutch residents in assisted housing and those in supported independent living revealed that the former had more service needs (met as well as unmet) than those in supported independent living although the level of impairment measured by HoNOS and subjective quality of life did not differ significantly between them [13].

In Germany, Richter [14] examined 1486 users of housing services in the region of Westphalia in northern Germany. He included housing services comprising closed inpatient facilities, open inpatient facilities, foster family care, and outpatient supported care settings. Richter found that the level of institutionalisation was positively related to the level of impairment measured by HoNOS but that the subjective quality of life was similar in all groups. However, the author noted that the mean HoNOS scores in the German sample were higher than those in the samples from Italy [14]. This is also true for the comparison of samples from the Netherlands [12, 13]. In terms of resident mobility between different levels of care, Richter revealed that about 80 % of the residents in the sample were assessed by staff members as being unable to live in a setting with an institutional level less than that currently provided [14].

Although the results of all the studies discussed above indicate that the level of institutionalisation in residential care is positively related to the level of impairment, no study has compared the level of impairment between patients in residential care with that of patients who live more independently in different housing settings in the community.

### Objectives

The main objective of this study was to examine to what extent the type of housing support received by patients with severe mental illness treated in outpatient clinics [15] is related to the severity of their mental illness, the impairment of their functional level, and their use of psychiatric treatment services.

## Method

### Study sample

Data were collected in a rural catchment area in southern Germany. Psychiatric in- and outpatient services for the catchment are provided by one psychiatric hospital with 180 beds and an outpatient clinic which treats about 1000 patients annually [16, 17].

Data were obtained from the patient record files of the regional Bavarian Outpatient Clinic Documentation System (AmBado), the Psychiatric Hospital Information System and from the regional social welfare agency. For the analyses, we first selected the records of all existing patients (*n* = 200) who received any type of housing support in the index-year 2010. This sampling procedure included patients who came to the outpatient clinic as well as those who were visited in their place of residence. All patients in this group had a Global Assessment of Functioning (GAF) score of 50 and below, indicating that they were not able to work on a regular basis and that they needed formal or informal support to cope with daily life activities. As a comparison group we consecutively selected the first 50 patients who consulted the outpatient clinic in 2010 and who fulfilled the criteria of severe mental illness (see below) but who received no housing support in the index-year. By this selection procedure we made sure that only patients with a similar level of functional impairment were compared in our analyses.

### Inclusion criteria

Patient records were included if the patients fulfilled the following criteria: 1) a GAF score equal to or below 50, 2) not able to work regularly due to his or her mental illness, and 3) receiving formal or informal support to cope with daily activities due to mental illness.

### Selection of study variables and assessment

#### Severity of mental disorder

The selection of study variables was limited by the information recorded in AmBado and the Psychiatric Hospital Information System (KIS). The only clinical measures provided by the AmBado were the General Assessment of Functioning (GAF) and the Clinical Global Impression (CGI) scales. Both of these quantitative assessments were conducted by the treating psychiatrists on an annual basis. In addition to the GAF and the CGI to indicate illness severity, we also considered a suicide attempt during lifetime, the existence of legal guardianship, and the cumulative length of psychiatric hospital stays during the indexed year. In a multilevel analysis of the determinants of GAF, Urbanoski et al. demonstrated, after controlling for patient-level predictors that only 7 % of the residual variance in admission GAF and 8 % of the residual variance in change scores was accounted by assessments of an individual patient made by different psychiatrists; these results lend support to the utility of GAF for drawing comparisons between patients sampled from a large institution [18].

#### Type of housing support

Information about housing support is provided by the AmBado. The following types of housing support were identified for the catchment area:

1. **No housing support:** Patients living in their own households (rental flats, apartments or public housing) and receiving psychiatric outpatient treatment by the outpatient clinic. Face-to-face care is given for approximately five hours per trimester by a mental health team.
2. **Outpatient housing support:** Patients living in their own households and receiving outpatient support from nurses and social workers (face-to-face contact) approximately one to five hours per week (maximum 10 h per week), in addition to the psychiatric outpatient care provided by the clinic.
3. **Foster family care:** Patients living in foster families for adults with additional support for both carer and patient provided by a team of social workers. The carer-to-patient ratio is 1:10, independent of psychiatric outpatient clinic provision.
4. **Group homes with 12-h staff:** Patients living in apartments with three to six residents with staff presence from 10 to 12 h a day.
5. **Intensively staffed group homes with 24-h staff:** Patients living in group homes with staff on duty round the clock. There are a maximum of 10 residents with a proportion of eight carers to 10 residents (ratio 1:1.25).
6. **Psychiatric nursing homes:** Patients living in psychiatric nursing homes with 50 to 70 residents and with 24-h nursing staff. The carer-to-patient ratio is about 1:4.

#### Costs of psychiatric treatment and psychosocial care

Cost data were obtained from AmBado, KIS, and the regional social benefit agency (Sozialverwaltung des Bezirks Schwaben). Costs of psychiatric in- and outpatient treatment and costs for psychotropic medication were individually calculated on systematic records by the health insurance prescription program (KV-Medikamentenverordnung-Modul). Costs of housing services were calculated as an average for each type of setting.

### Privacy and data protection

Patient identification codes were used to link the information from the AmBado and from KIS. After linking the data sets, the data file was anonymised by dropping the patient identification code from the data file.

### Ethical considerations

According to German statutes, informed consent is not needed for the use of pseudonymised patient record data. The current investigation was approved by the ethics committee of the Bavarian Medical Association (EK-Nr. 2014-071).

### Statistical analyses

Bivariate statistical associations between patient characteristics and type of housing support were tested by the Pearson Chi^2^ test in case of categorical variables or analysis of variance (ANOVA) with post-hoc test corrected for unequal variances (Scheffé) in case of continuous variables.

The multivariate association of GAF and CGI with type of housing service was analysed by means of linear multiple regression analyses controlling for diagnosis, age, gender and living situation. Associations of housing services with suicide attempts and having a guardian were analysed by means of logistic regression analyses controlling for diagnosis, gender, age and living situation. In multivariate linear regression models as well as in logistic regression models, type of housing is coded as a group variable with six values, one of them (“no housing support”) is considered as reference category (Tables 3, 4 and 5).

Associations of costs of psychiatric and psychosocial care with type of housing service use were analysed by means of linear regression analyses. Robust standard error estimation was applied to take into account the skew distribution of cost data.

Post hoc power analyses for linear regression models indicated that an effect size of f^2^ = 0.045 could be obtained at *p* = 0.05 with a power of 95 %. Post hoc power analyses for logistic regression models indicated that an odds ratio of 1.74 respective of 0.54 could be obtained at *p* = 0.05 with a power of 95 %. Statistical analyses were performed using Stata 13; power analyses were performed using G-power 3.1.5.

## Results

In the patient sample, 50 (20 %) lived in their own households in the community without any kind of housing support. Out of the 200 patients who received outpatient housing support 41 (16.4 %) lived in their own households in the community and received ambulant housing support from nurses and social workers, 23 (9.1 %) lived in foster families for adults, 45 (18 %) lived in group homes with staff support during the day, 10 (4 %) lived in group homes with 24 h staff support and 81 (32.4 %) lived in nursing homes with 24 h staff support, as can be seen in Table 1.

**Table 1:** Descriptive statistics for patient sample by housing setting

	Total (*N* = 250)	(1) Private household without welfare support (*N* = 50)	(2) Private household with welfare support (*N* = 41)	(3) Foster families for adults (*N* = 23)	(4) Staffed apartments (*N* = 45)	(5) Intensively staffed group homes (*N* = 10)	(6) Psychiatric nursing homes (*N* = 81)	Tests (Pearson Chi2 or ANOVA with Scheffé test))
Gender: female n (%)	84 (34 %)	17 (34 %)	15 (37 %)	10 (43 %)	16 (36 %)	3 (30 %)	23 (28 %)	Chi2 (5) = 2.29
								p = 0.807
Age M (SD)	53.7 (13.1)	51.7 (10.5)	49.4 (11.6)	61.9 (14.8)	48.7 (14.2)	51.5 (6.4)	57.8 (12.7)	F = 6.49
								p < 0.001
								3 > 2,4; 4 < 6
Living situation: living alone n (%)	236 (94.4 %)	42 (84 %)	39 (95 %)	23 (100 %)	43 (95 %)	10 (100 %)	79 (97.5)	Chi2 (5) = 13.84
								p = 0.017
Diagnosis of Schizophrenia n (%)	158 (63 %)	27 (54 %)	25 (61 %)	14 (61 %)	37 (82 %)	9 (90 %)	43 (57 %)	Chi2 (5) = 13.5
								p = 0.019
GAF M (SD)	31.62 (8.47)	31.1 (6.84)	30.5 (8.98)	30.4 (5.71)	29.29 (7.68)	28.2 (3.05)	34.57 (9.86)	F = 3.41
								p = 0.005
								4 < 6
CGI M (SD)	7.06 (0.79)	7.18 (0.56)	6.9 (0.89)	6.74 (0.69)	7.16 (0.56)	7.1 (0.32)	7.09 (0.99)	F = 1.31
								p = 0.260
Average number of hospitalisations during index year; M (SD)	0.4 (1.16)	0.24 (0.55)	0.34 (0.76)	0.22 (0.67)	0.53 (1.9)	0.6 (1.08)	0.47 (1.21)	F = 0.55
								p = 0.737
Cumulative LOS during index year; M (SD)	9.88 (35.1)	9.3 (30.6)	10.4 (40.6)	14.8 (53.6)	12.7 (46.3)	6.8 (16.2)	7.37 (20.56)	F = 0.25
								p = 0.940
Current legal guardianship n (%)	189 (76 %)	18 (36 %)	35 (85 %)	19 (83 %)	33 (73 %)	9 (90 %)	75 (93 %)	Chi2 (5) = 59.17
								p < 0.001
Confirmed suicide attempt during lifetime n (%)	120 (48 %)	30 (60 %)	18 (44 %)	4 (17 %)	24 (53 %)	6 (60 %)	38 (47 %)	Chi (5) = 12.92
								p = 0.024

Descriptive statistics: For continuous variables: M (mean), SD (standard deviation); for categorical variables: sample size (percentages)

Tests: Pearson Chi-square for categorical variables; ANOVA (Scheffé including Bartlett’s correction) for continuous variables. Chi2 () = Value on Chi2-distribution for given degrees of freedom (); F = value on F-distribution for variances; p = level of significance

Dichotomous variables: gender: women = 0; men = 1; living situation: 0 = single; 1 = living in couple; diagnosis: 1 = schizophrenia; 0 = any other diagnosis according to ICD-10; Current legal guardianship: 0 = no guardianship; 1 = underlying a guardianship; Certain suicide attempt lifetime: 0 = not or not certain; 1 = certain at least one suicide attempt during lifetime

Abbreviations: *GAF* Global Assessment of Functioning, *CGI* Clinical Global Impression, *LOS* Length of in-patient stay

The comparison of patient characteristics indicated no significant gender distributions between the housing support groups. Patients living in foster families and those living in psychiatric nursing homes were older than those who lived in their own households with ambulant housing support or in staffed apartments. Most of the patients lived alone and no statistical differences in these basic variables were found between the different housing types. The share of patients with a diagnosis of schizophrenia was highest in staffed apartments and high staffed group homes.

The lowest GAF scores were obtained for patients living in high staffed apartments (m = 28.2; sd = 3.05) and in staffed apartments (m = 29.20; sd = 7.68) while the highest GAF score was found for patients in psychiatric nursing homes (m = 34.57; sd = 9.86). However, significant differences in GAF score were only found between patients living in nursing homes and those who lived in staffed apartments. No significant differences were found for the CGI scores, the average number of hospitalisations or the cumulative length of stay in psychiatric hospitals during the index-year. The proportion of patients with a legal guardianship was lowest (36 %) for those who lived in their own households without housing support and highest for those who lived in high staffed group homes (90 %) or nursing homes (93 %). The rate of lifetime suicide attempts was lowest in patients living in foster families (17 %) and highest in those who did not receive housing support (60 %) and those who lived in high staffed group homes.

The comparison of the psychiatric service costs across the different housing service groups (Table 2) indicates no significant differences in inpatient treatment costs or the costs of psychopharmacological treatment. Costs of outpatient treatment received by patients living in psychiatric nursing homes or in foster families were significantly lower compared to patients living in own households with outpatient housing support. Since only average housing cost data were available in the data set, no statistical test was performed to assess if cost differences existed between the housing groups. As might be expected, patients without housing services generated no housing costs and the average costs for housing services were highest in high staff group homes. The combined costs of housing and psychiatric treatment were highest in patients living in staffed apartments and lowest in patients without housing services. The proportion of housing costs to total costs varied between 79 and 92.5 %.

**Table 2:** Costs of psychiatric treatment and additional social support by type of housing settings

	(1) Private household without housing support (*N* = 50)	(2) Private household with ambulant housing support (*N* = 41)	(3) Foster families for adults (*N* = 23)	(4) Staffed apartments	(5) Intensively staffed group homes	(6) Psychiatric nursing homes (*N* = 81)	Test (ANOVA with Scheffé post hoc test)
				12 hrs. Staff (*N* = 45)	24 hrs. Staff (*N* = 10)		
Inpatient treatment costs (€)	2,278	2,546	3,632	3,120	1,666	1,819	F = 0.25
							p = 0.905
Outpatient treatment costs (€)	1,188	1,674	528	1,125	744	508	F = 5.47
							p = 0.001
							2 > 3,6
Psychopharmacological costs (€)	1,205	1,226	737	1,942	2,274	1,878	F = 2.70
							P = 0.021
Costs of psychiatric treatment (€)	4,672	5,446	4,898	6,186	4,684	4,255	F = 0.29
							p = 0.918
Housing costs (€)	-	22,400	18,485	23,390	57,450	27,500	
Total costs of psychiatric care (€)	4,672	27,846	23,383	29,576	62,134	31,755	F = 93.37
							p < 0.001
							1 < 2,3,4,6 < 5
Proportion of housing costs to total costs (%)	0	80 %	79 %	79 %	92.5 %	86.6 %	

Tests: ANOVA (Scheffé including Bartlett’s correction for inequal variances). F = Value on F-distribution for variances; p = level of significance

Results of the regression analyses (Table 3) indicate that patients living in psychiatric nursing homes have a GAF score that is 4.5 points (*p* = 0.002) higher compared to patients living in the community without any housing support. The R^2^ suggests that about 11 % of the GAF variance is explained by the model. The functional level of the patients in the other housing groups show no significant differences compared to those without any housing support. The regression coefficients for the CGI score indicate that patients living in foster families for adults have a lower severity of mental illness compared to those with no housing support (b = -0.52; p = 0.004), whilst the other housing support groups do not significantly differ from the reference group. The model explains about 6 % of the CGI variance.

**Table 3:** Linear Regression models for the association between clinical characteristics and accommodation setting

	GAF		CGI	
	b	p	b	p
Type of housing: “No housing support” as reference category				
Private household with add. welfare supp.	-0.433	0.799	-0.264	0.114
Foster families for adults	1.089	0.603	-0.520	0.012
Staffed apartments	-1.340	0.634	-0.188	0.494
Intensively staffed group homes	4.570	0.002	-0.129	0.379
Psychiatric nursing homes	-0.941	0.578	-0.108	0.513
Control variables				
Age	-0.117	0.005	0.002	0.613
Gender	-0.105	0.923	-0.144	0.176
Living situation (single)	2.098	0.364	-0.178	0.429
Diagnosis of schizophrenia	-3.467	0.002	0.238	0.029
Constant	38.762	0.000	7.069	0.000
F/p	4.47	0.000	2.23	0.021
R^2^	0.143		0.058	
N	250		250	

Abbreviations: *GAF* Global Assessment of Functioning, *CGI* Clinical Global Impression;

Statistical parameters: b = regression coefficient; p = level of significance; F = value on F-distribution for variances; R^2^ = fit of regression analysis as degree of explanation effect by this model; N = sample size

Dichotomous variables: gender: women = 0; men = 1; living situation: 0 = single; 1 = living in couple; diagnosis: 1 = schizophrenia; 0 = any other diagnosis according to ICD-10

The logistic regression model for suicide attempts (Table 4) indicates that patients living in foster families have an 89 % lower prevalence of attempting suicide than those who do not receive any housing support. The pseudo R^2^ reveals that about 8 % of the variance in the prevalence of suicide attempts is explained by the model. In contrast, the odds ratios for the existence of legal guardianship (Table 5) show that the probability of having a legal guardian for patients who receive one of the various forms of housing support is 5 to 18 times higher than in the group without housing support. As indicated by the pseudo R^2^, the logistic regression model explains about 29 % of the variance in the probability of having a legal guardianship.

**Table 4:** Logistic Regression models for the association between suicide attempts, legal guardianship and accommodation setting

	Suicide attempt during lifetime		Legal guardianship	
	OR	p	OR	p
Type of housing: “No housing support” as reference category				
Private household with add. welfare supp.	0.450	0.074	13.665	0.000
Foster families for adults	0.112	0.001	4.779	0.017
Staffed apartments	1.010	0.989	11.907	0.026
Intensively staffed group homes	0.562	0.143	18.831	0.000
Psychiatric nursing homes	0.717	0.454	5.708	0.001
Control variables				
Age	0.994	0.558	1.060	0.000
Gender	0.398	0.002	1.090	0.824
Living situation (single)	0.434	0.174	0.122	0.008
Diagnosis of schizophrenia	0.737	0.299	1.686	0.177
Constant	5.344	0.016	0.023	0.000
F/p	27.01	0.000	79.69	0.000
Pseudo R^2^	0.078		0.287	
N	250		250	

Abbreviations: *GAF* Global Assessment of Functioning, *CGI* Clinical Global Impression;

Statistical parameters: OR = odds ratio; p = level of significance; F = value on F-distribution for variances; Pseufo R^2^ = fit of regression analysis as degree of explanation effect by this model; N = sample size

Dichotomous variables: gender: women = 0; men = 1; living situation: 0 = single; 1 = living in couple; diagnosis: 1 = schizophrenia; 0 = any other diagnosis according to ICD-10

**Table 5:** Linear Regression models for the association between cost of psychiatric care and accommodation setting

	Cost of psychiatric inpatient treatment		Costs of psychiatric outpatient treatment		Costs of psychopharmacological treatment		Total costs of psychiatric care without housing service costs		Total costs of psychiatric care including housing service costs	
	b	p^1^	b	p^1^	b	p^1^	b	p^1^	b	p^1^
Type of housing	No housing support : Reference category									
Ambulant housing support	1694.71	0.405	408.25	0.335	-161.14	0.694	1939.03	0.377	24339.03	0.000
Foster families for adults	3918.69	0.269	-703.25	0.000	-387.60	0.230	2804.15	0.437	21289.15	0.000
Staffed apartments	1104.28	0.597	-515.69	0.004	613.50	0.526	1193.23	0.577	58643.23	0.000
Intensively staffed group homes	1250.59	0.440	-776.70	0.000	598.48	0.120	1121.95	0.510	28621.95	0.000
Psychiatric nursing homes	2177.02	0.335	-102.10	0.604	379.10	0.368	2447.80	0.286	25837.80	0.000
Control variables										
Age	-41.76	0.217	-0.40	0.924	-15.50	0.130	-56.71	0.125	-56.71	0.125
Gender	-783.86	0.561	-233.65	0.217	289.93	0.182	-711.79	0.610	-711.79	0.610
Living situation	-3570.19	0.011	-555.34	0.008	-403.79	0.136	-4536.11	0.002	-4536.11	0.002
Diagnosis of schizophrenia	-2433.21	0.205	136.90	0.410	191.01	0.585	-2112.06	0.294	-2112.06	0.294
Legal guardianship	3448.81	0.002	263.36	0.077	236.46	0.318	3912.36	0.001	3912.36	0.001
Suicide attempt	-2646.25	0.046	-226.25	0.271	756.77	0.001	-2085.08	0.134	-2085.08	0.134
Constant	5763.48	0.033	1366.78	0.000	1261.16	0.033	8340.43	0.003	8340.43	0.003
F/p	1.35	0.197	5.28	0.000	3.85	0.000	1.96	0.334	140.29	0.000
R^2^	0.11		0.14		0.11		0.10		0.69	
N	250		250		250		250		250	

Statistical parameters: b = regression coefficient; p^1^ Robust estimation of standard errors; F = value on F-distribution for variances; R^2^ = fit of regression analysis as degree of explanation effect by this model; N = sample size. Dichotomous variables: gender: women = 0; men = 1; living situation: 0 = single; 1 = living in couple; diagnosis: 1 = schizophrenia; 0 = any other diagnosis according to ICD-10

Regression models for the cost data (Table 5) reveal that the psychiatric inpatient treatment costs and the costs for psychopharmacological treatment are not related to the type of housing service at all, whilst the expenditures for psychiatric inpatient treatment is significantly lower for patients living in foster families (b = -703.25; p = 0.000), staffed apartments (b = -515.69; p = 0.004) or high staffed group homes (b = -776.70) compared to those who live in private households without housing support. The total costs of psychiatric treatment without housing service costs are not related to the type of housing service but the total costs of mental health care including the housing service costs are significantly higher for patients who receive any type of housing support.

## Discussion

To our knowledge, this is the first study that compares the severity of mental illness between psychiatric patients with different levels of housing support, which also includes patients who live in the community without any type of housing support.

Results of our study revealed that the type of housing service received by the patients was not related to the indicators of illness severity as expected. We found that patients who lived in nursing homes had a higher level of functioning and patients who lived in foster families for adults had a lower severity of illness than those who lived independently in the community without any kind of housing support. The proportion of patients with a diagnosis of schizophrenia was highest in residents of staffed apartments and high staffed group homes but varied only slightly between patients who did not receive any housing support and those who received ambulant housing support or lived in foster families or in nursing homes. On the other hand, the type of housing support was clearly related to the existence of a legal guardianship. Patients who received housing support had a 5 to 18 fold probability of having a legal guardianship compared to those who received no housing support.

Legal guardianship (US Psychiatry distinguishes between “guardianship” and “conservatorship” depending on the emphasis of tasks) is a very special and characteristic German feature. Legal guardianship has existed for more than a century and the system aims to protect the legal rights of people suffering from severe mental disorders that are faced with difficulties in maintaining autonomy and the risk of relapses which might lead to rehospitalisation. Guardianships can only be ordered by courts for specific reasons (normally financial tasks, commitment authorization, compliance with treatment, help in housing difficulties, etc.). A middle and long-term guardianship can only be ordered on the basis of a detailed psychiatric assessment. Guardianship Law is included in the German Civil Law Code. After the Reform Act of Guardianship Law 1992, the number of guardianships as well as the number of involuntary admissions covered by guardianships increased dramatically. In Germany, there are currently about 1.4 million people assigned guardianship, corresponding to 1.7 % of the population. This internationally high rate of guardianships [19] is associated with an augmentation of compulsory admissions [4, 20, 21].

Due to the cross-sectional nature of our study, it is not possible to make conclusions about the causal nature of the obtained effects. So it is not possible to say whether the implementation of legal guardianship causes a higher level of institutionalisation. From everyday mental health practise, we know that, in some cases, the legal guardian initiates the admission of the patient to a higher type of institutional housing service; in other cases, staff at the residential facility initiates the establishment of a legal guardian, as previously discussed by the authors [22].

Our results are in accordance with the findings of Shepherd and Murray [1] who note that, in general, evidence favouring the relative effectiveness of different community housing settings is weak and there seem to be few differences in terms of clinical functioning, social functioning, or readmission risk among different forms of shelter and supported housing. However, most residents prefer facilities that are small and offer a greater degree of privacy and independence [1].

While it was not possible to trace back the level of housing support to clinical or functional differences, our results make obvious that institutional level has significant effects on the total cost of mental health care. Our detailed cost analyses revealed that a higher level of supported housing was related to increased total costs of mental health care but to decreased expenditures for psychiatric outpatient treatment, while the level of housing support was not related to costs of inpatient treatment or costs of outpatient medication. These associations reinforce our suspicion that the level of housing support in our sample is not adequately justified by the clinical or functional status of the patients. Moreover, the negative association between the level of housing support and the expenditures for outpatient treatment might suggest that a higher level of housing support could be related to an inadequate provision of psychiatric outpatient treatment. Either because the need for psychiatric treatment is not adequately recognized in more institutionalized settings, or because inadequate provision of outpatient care increases the patients’ risk to end up in a more institutionalized setting.

It is surprising that psychiatric nursing homes show the same proportion of individuals suffering from schizophrenia as the sample drawn from supervised apartments, since we expected a higher degree of institutionalization in patients suffering from chonic psychotic illnesses due to their higher support needs. Considering a prior longitudinal investigation of the authors on a psychiatric nursing home [22], the switch from a public to a private stakeholder may play an important role for admission rationale accounting for diagnosis.

Due to the cross-sectional character of our study it is not possible to test causal hypotheses. Nevertheless, our results make obvious that the process of housing support implementation must be further investigated to understand whether the identified mismatch between clinical characteristics and the type of housing support is caused by a general lack of evidence based guidelines for psychosocial mental health care or by the lack of adequate mental health service provision.

## Conclusions

These findings suggest that the accommodation and welfare needs of severe psychiatric impaired patients are not adequately reflected both in the measures of severity and functional impairment and in the use of psychiatric treatment resources. In particular, the type and the costs of accommodation services are not adequately related to the severity of the mental disorder and to the needs of the mentally ill patients [23]. Independently of these results, it is necessary to clarify what criteria are being used to decide on an appropriate housing setting, with respect to the extent of welfare or social support that takes place in the routine care of severe mentally ill patients. One possible positive explanation could be that the decision is based on a very careful consideration of the patient’s individual needs and includes his or her personal wishes. Some possible negative interpretations are that the decision is based on the availability of housing settings, the strength of accommodation facilities marketing strategies, or for solely economic reasons, without taking the patients’ needs or wishes into account.

Given the fact that an inadequate level of institutionalisation in mental health care causes on the one hand unnecessary restricts patients’ freedom and on the other hand adds unnecessary costs to society, it seems essential to investigate which of these explanations is true, especially because of the challenge of dealing with adequate care of new long-term residential patients [24, 25].

Due to the limited size of our database, it is not possible to answer this question here. An adequate study design should include a prospective, in-depth analysis of the decision-making process for choosing different types of residential care in routine practice.

### Limitations

This study has several limitations. First, we used only data from patients’ electronic records. Therefore, we could include only variables which were available in the AmBado system to explain the variance in the use of housing services. Due to this limitation only global measures of illness severity such as the GAF and the CGI were available.

Second, we used only data from one catchment area. Therefore, our results are not representative of the situation in Bavaria or Germany more broadly [17]. To account for the heterogeneity of mental health care services across Germany, an adequate study should compare a representative sample of patients from different federal states. In addition, a larger sample size would allow the application of more advanced statistical analyses, such as multinomial logistic regression models, which would be more adequate for the investigation of processes with multi-categorical target variables, such as type of housing.

Third, due to our cross-sectional study design, no conclusions about the causal direction of the revealed associations are possible. Longitudinal data is necessary to get a better understanding of the reasons for transition between different types of housing in correspondence with patients’ needs profiles.

### Implications for clinical practice

In order to make sure that housing support for persons with severe mental illness becomes more closely related to the needs and wishes of the patients, it is necessary to make decisions about the adequate type of housing support on the basis of systematic assessment of needs. Furthermore it also seems necessary to review the fit between the housing setting and the clinical status of the patient on a regular basis to make sure that changes in patients’ needs and wishes will be adequately considered when decisions about psychiatric service are made.

More detailed assessments of patients’ impairments, of their objective and subjective support needs, and of their social and economic living circumstances are needed; likewise, also more information about the decision-making criteria applied by the mental health care staff and respective stakeholders in the selection of the adequate type of housing would be needed.

## Abbreviations

AmBADO: Ambulante Basisdokumentation (The mandatory Bavarian Outpatient Clinic Documentation System)
b: Regression coefficient
CAN: Camberwell Assessment of Need
CGI: Clinical Global Impression Scale
EK-Nr: Processing code by the ethics committee
f^2^: Population effect size
GAF: Global Assessment of Functioning Scale
HoNOS: Health of the Nation Outcome Scale
KIS: Krankenhausinformationssystem (IT-Hospital Information System)
KV: Kassenärztliche Vereinigung (Association of Statutory Health Insurance Physicians)
N: Sample size
OR: Odds ratio
p: Level of significance
R^2^: Proportion of the variance explained by the regression model
SD: Standard deviation
SMI: Severe mental Illness
UK: United Kingdom
